# PFCs and Cholesterol: A Sticky Connection

**DOI:** 10.1289/ehp.118-a81b

**Published:** 2010-02

**Authors:** Tanya Tillett

**Affiliations:** **Tanya Tillett**, MA, of Durham, North Carolina, is a staff writer/editor for *EHP*. She has been on the *EHP* staff since 2000 and has represented the journal at national and international conferences

Polyfluoroalkyl chemicals (PFCs), highly stable compounds used in consumer items such as food packaging, textiles, and paper products, are known to migrate throughout and persist in the environment. Animal studies have described the development of adverse health effects such as tumors and developmental delays with exposure to the PFCs perfluorooctanoic acid (PFOA) and perfluorooctane sulfonic acid (PFOS). A new study now suggests these and other PFCs may affect serum cholesterol levels in humans **[*EHP*** **118:197–202; Nelson et al.]**.

Elevated cholesterol levels are associated with an increased risk of cardiovascular disease, and are one of the conditions that define metabolic syndrome. Risk factors for high cholesterol include diet, low physical activity, and a family history of the condition, but increasing evidence indicates some environmental chemicals also may contribute. With estimated half-lives of up to 8.5 years, PFCs are classified as persistent organic pollutants (POPs). However, unlike most POPs, which are stored primarily in fat tissue, PFCs persist by forming chemical bonds to proteins in the liver and serum.

Previous studies in humans have reported positive associations between PFOS and PFOA exposures and higher cholesterol levels. The research team in the current study investigated the relationship between insulin resistance, cholesterol levels, body size, and exposure to two less studied PFCs, perfluorononanoic acid (PFNA), and perfluorohexane sulfonic acid (PFHxS) in addition to PFOS and PFOA. The study used data from the 2003–2004 National Health and Nutrition Examination Survey.

The authors found a positive association between total cholesterol (TC) and serum concentrations of PFOS, PFOA, and PFNA. TC is the sum of low-density and very low-density lipoproteins (“bad” cholesterol) and high-density lipoproteins (“good” cholesterol). The findings appeared driven by an increase in the non–high-density lipoprotein fraction of TC, and the association was most pronounced for PFNA. In contrast, PFHxS concentrations were inversely related to TC: samples reflecting the highest PFHxS exposure had the lowest TC levels.

The authors observed little evidence of associations between body size, insulin resistance, and PFC concentrations. They note several limitations in this study, including the fact they could not rule out the possibility of reverse causality—that is, that having higher cholesterol levels could lead to increased PFC concentrations in the blood. Nevertheless, the results support previous epidemiologic research indicating that environmentally relevant exposures to PFCs may affect human cholesterol metabolism or homeostasis.

## Figures and Tables

**Figure:**
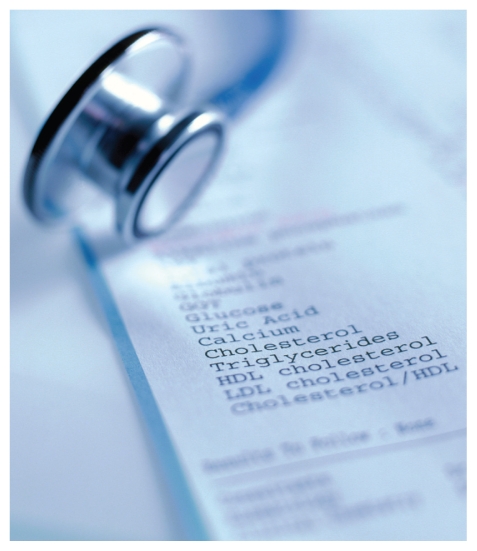
Certain environmental chemicals may be a risk factor for high cholesterol.

